# The association between the neutrophil-to-lymphocyte ratio and type 2 diabetes mellitus: a cross-sectional study

**DOI:** 10.1186/s12902-024-01637-x

**Published:** 2024-07-09

**Authors:** Hai long Chen, Chunwei Wu, Lei Cao, Ruolin Wang, Tian yang Zhang, Ze He

**Affiliations:** 1 Changchun University of Traditional Chinese Medicine, 1035 Bo shuo Road, Changchun, Jilin 130117 China; 2 Department of Endocrinology, The Affiliated Hospital of Changchun University of Traditional Chinese Medicine, 1478 Gong nong Road, Changchun, Jilin 130021 China

**Keywords:** Neutrophil-to-lymphocyte ratio, Inflammation, Type 2 diabetes mellitus, Cross-sectional study

## Abstract

**Background:**

Type 2 diabetes mellitus (T2DM) is a prevalent chronic disease often accompanied by low-grade inflammation. Recently, the neutrophil-to-lymphocyte ratio (NLR) has garnered researchers’ interest as an emerging inflammation biomarker. This study aimed to comprehensively explore the relationship between NLR and T2DM using the National Health and Nutrition Examination Survey (NHANES) database.

**Method:**

We employed a cross-sectional study design to analyze data from five NHANES cycles from 2007 to 2016, excluding individuals with incomplete data. This study utilized a weighted logistic regression model, subgroup analyses, and restricted cubic spline (RCS) analysis to assess the potential relationship between NLR and T2DM.

**Results:**

A total of 9903 participants were eligible for the analysis, of which 1280 were diagnosed with T2DM. The T2DM group exhibited significantly higher NLR levels than the non-T2DM group. After adjusting for potential confounders, elevated NLR levels were associated with an increased risk of developing T2DM, indicated by an odds ratio (OR) of 1.14, 95% CI: (1.05,1.24), *P* = 0.003. The results of the subgroup analyses revealed a significant interaction effect between NLR and T2DM concerning race and hypertension (*P* for interaction < 0.05). In contrast, no significant interactions were found for age, sex, education level, body mass index (BMI), smoking status, recreational activities, and alcohol drinker (*P* for interaction > 0.05). RCS analysis showed a significant non-linear relationship between NLR and T2DM, with an inflection point at 2.27 (all *P* for non-linearity < 0.05).

**Conclusion:**

Our study indicates that an elevated neutrophil-to-lymphocyte ratio is associated with a higher risk of T2DM.

**Supplementary Information:**

The online version contains supplementary material available at 10.1186/s12902-024-01637-x.

## Introduction

Type 2 diabetes mellitus (T2DM), one of the most serious and prevalent chronic diseases, can lead to a variety of complications such as cardiovascular diseases, nephropathy, neuropathy, retinopathy, lower limb amputations, and reduced life expectancy, significantly contributing to increased mortality rates in T2DM patients [1–3]. The prevalence of T2DM is rising with the rapid development of the global economy and lifestyle changes. Epidemiological data indicate that, as of 2021, the global prevalence of T2DM among individuals aged 20–79 years was estimated at 10.5% (536.6 million people), with projections suggesting that over 1.31 billion people worldwide could be affected by 2050, with similar rates observed in both genders [4].

The primary clinical criterion for diagnosing T2DM is elevated venous blood glucose levels. T2DM comprises 90% of all cases of diabetes mellitus [5]and is associated with several pathogenic factors, including genetic predispositions, immunological factors, environmental influences, insufficient physical activity, and poor lifestyle choices [6–8]. The pathogenesis primarily involves the relative insufficiency of insulin secretion by pancreatic β-cells and the insensitivity of tissues and organs to insulin, which triggers insulin resistance (IR). This leads to a compensatory increase in insulin secretion, ultimately causing pancreatic β-cell damage and failure [9, 10].

The role of inflammation in the development of T2DM and associated metabolic disorders has garnered significant attention [11, 12]. In recent years, the NLR has been increasingly studied as a composite biomarker that better reflects the systemic inflammatory state compared to individual biomarkers, being cost-effective and easy to detect [13–15]. NLR has been reported as a reliable inflammatory marker in type 2 DM [16] and other inflammatory conditions including gastrointestinal diseases [17], cardiac conditions [18], thyroiditis [19], thyroid conditions [20], irritable bowel disease [21], and Covid-19 infection [22]. Hence, studying the association between T2DM and NLR is reasonable. However, previous studies have been constrained primarily by their small sample sizes, leaving the relationship between NLR and T2DM ambiguous. Consequently, this study aims to elucidate the potential relationship between NLR and T2DM using a large dataset from the NHANES, seeking to uncover new insights.

## Methods

### Participant selection and process

**Fig. 1:**
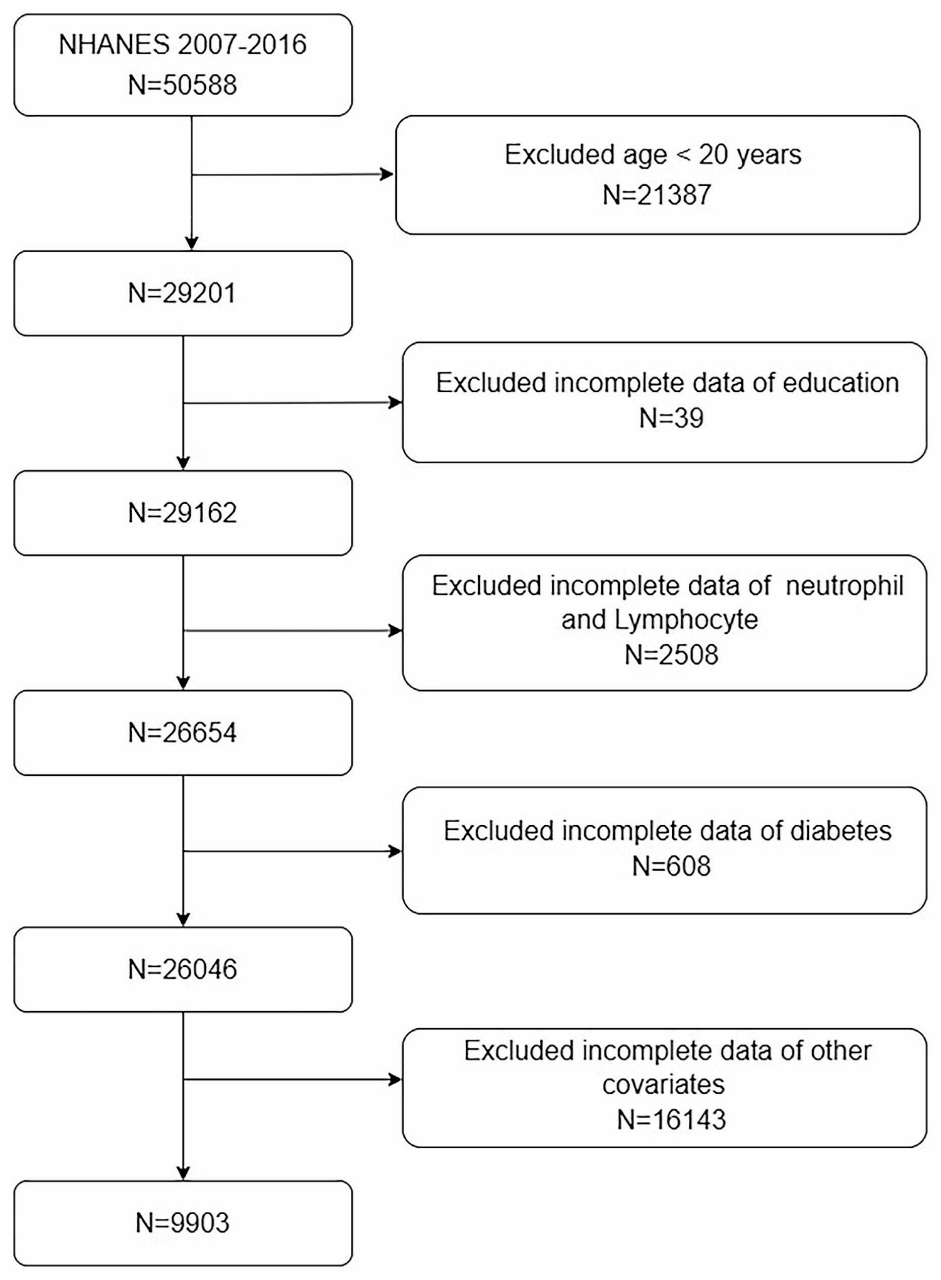
Participants and flowcharts

The NHANES is a population-based, cross-sectional survey conducted by the Centers for Disease Control and Prevention (CDC) to assess the health and nutritional status of adults and children in the United States. The research team includes professional health investigators, medical technicians, and physicians. The NHANES database, updated biennially, comprises demographic, dietary, examination, laboratory, and questionnaire data. All participant data collection was conducted with informed consent and approved by an ethical review board. We used data from 2007 to 2016 to select participants. We initially screened 50,588 participants, with the specific exclusion criteria as follows: Exclude participants < 20 years old (*n* = 21,387); Exclude participants lacking education data (*n* = 39); Exclude participants missing neutrophil and lymphocyte counts (*n* = 2,508); Exclude participants missing diabetes data (*n* = 608); Exclude participants lacking other important covariate data (*n* = 16,143). A total of 9,903 eligible participants were included (Fig. 1).

### Detection and definition of NLR

Venous blood was collected in the morning after an overnight fast at the Mobile Examination Centre, and a Beckman Coulter DxH 800 instrument was utilized to perform a complete blood count on the specimen. NLR was determined by dividing the count of neutrophils by that of lymphocytes [23].

### Definition of T2DM, hypertension

Diagnosis of T2DM was established based on: (1) self-report of T2DM, (2) fasting blood glucose ≥ 7.0 mmol/L, (3) presence of T2DM symptoms with random blood glucose ≥ 11.1 mmol/L, (4) glycosylated hemoglobin A1c (HbA1c) ≥ 6.5% [24]. Diagnostic criteria for hypertension: NHANES participants were surveyed by healthcare professionals both at home and at the Mobile Examination Center. In the questionnaire, participants were asked, “Have you ever been told you have high blood pressure?” Response options were “Yes” or “No.” Participants answering “Yes” were classified as having hypertension, and those answering “No” were classified as not having hypertension.

### Covariates

The covariates comprised demographic, anthropometric, and laboratory measures. The covariates were specified as follows: age groups (20–39, 40–59, and ≥ 60 years), sex (male and female), racial categories (Mexican American, other Hispanic, non-Hispanic white, non-Hispanic black, and other races), education levels (less than high school, high school, or higher), and BMI, classified into three categories: normal, overweight, and obese (< 25 kg/m^2^, 25–29.9 kg/m^2^, and ≥ 30 kg/m^2^). Smoking status was categorized as Never, Former, or Current. Participants were queried regarding whether they had ever smoked 100 cigarettes in their lifetime and if they were currently smoking to distinguish between current and former smokers. Participants were classified as never smokers if they had consumed fewer than 100 cigarettes in their lifetime. Participants were classified as ex-smokers if they were not current smokers but had consumed 100 cigarettes in the past. The activity was defined as any moderate-intensity exercise, fitness, or recreational activity leading to a slight increase in breathing or heart rate-such as brisk walking, bicycling, swimming, or volleyball for at least ten consecutive minutes weekly. Drinkers were defined as individuals who consumed at least 12 alcoholic beverages annually. Additionally, we included hypertension, poverty income ratio (PIR), Total cholesterol (TCHO), triglyceride (TG), Low-density lipoprotein cholesterol (LDL-C), High-density lipoprotein cholesterol (HDL-C), HbA1c. All covariates were sourced from the NHANES database.

### Statistical analysis

DecisionLinnc1.0 software was employed for data analysis [25]. DecisionLinnc1.0 is a platform that integrates multiple programming language environments and enables data processing, data analysis, and machine learning through a visual interface. We refer to the “WTMEC2YR” weighting variable and multiply the 2-year MEC weights by one-fifth to derive 10-year weights. Categorical variables are expressed as percentages, Continuous variables are first tested for normality. Data following a normal distribution are represented by Mean ± Standard Deviation, while data not following a normal distribution are often described by the median and interquartile range to depict the central tendency and dispersion. Weighted logistic regression was employed across three distinct models to examine the relationship between NLR and T2DM. Model 1 was not adjusted for covariates. In Model 2, adjustments were made for age, sex, and race. Model 3 included adjustments for sex, age, race, education level, BMI, smoking status, recreational activities, alcohol drinker, hypertension, PIR, TCHO, TG, LDL-C, HDL-C, and HbA1c. Subgroup analyses were also conducted. Furthermore, RCS was utilized to explore potential non-linear relationships between NLR and T2DM risk. *P* < 0.05 was considered statistically significant.

## Results

### The characteristics of the participants

A total of 9,903 participants with complete data were included in this analysis (Fig. 1). Of these, 8,623 were non-T2DM, and 1,280 were T2DM. Compared to the normoglycemic group, the diabetic group was older (*P* < 0.001), better educated (*P* < 0.001), exhibited a higher obesity rate (*P* < 0.001), and a greater prevalence of individuals who lacked regular exercise and alcohol drinker (*P* < 0.001). The differences in PIR, TCHO, TG, LDL-C, HDL-C, and HbA1c between the two groups were statistically significant (*P* < 0.001). Detailed information can be found in Table 1.

**Table 1:** The characteristics of the study participants

Variable Names	Overall	Non-T2DM	T2DM	*P*
n	9903	8623	1280	
Age (%)				
20-39	3491 (35.25)	3404 (39.48)	87 (6.80)	<0.001
40-59	3095 (31.25)	2685 (31.14)	410 (32.03)	
≥ 60	3317 (33.49)	2534 (29.39)	783 (61.17)	
Sex (%)				
Male	4847 (48.94)	4188 (48.57)	659 (51.48)	0.055
Female	5056 (51.06)	4435 (51.43)	621 (48.52)	
Race (%)				
Mexican American	1480 (14.94)	1266 (14.68)	214 (16.72)	<0.001
Other Hispanic	1028 (10.38)	876 (10.16)	152 (11.88)	
Non-Hispanic White	4512 (45.56)	4018 (46.60)	494 (38.59)	
Non-Hispanic Black	1898 (19.17)	1574 (18.25)	324 (25.31)	
Other Race	985 (9.95)	889 (10.31)	96 (7.50)	
Education level (%)				
< High school	2382 (24.05)	1934 (22.43)	448 (35.00)	<0.001
≥High school	7521 (75.95)	6689 (77.57)	832 (65.00)	
BMI (%)				
<25	2952 (29.81)	2783 (32.27)	169 (13.20)	<0.001
25-29.9	3310 (33.42)	2946 (34.16)	364 (28.44)	
≥30	3641 (36.77)	2894 (33.56)	747 (58.36)	
Smoking status (%)				
Never	5447 (55.00)	4785 (55.49)	662 (51.72)	<0.001
Former	2437 (24.61)	2020 (23.43)	417 (32.58)	
Current	2019 (20.39)	1818 (21.08)	201 (15.70)	
Recreational activities (%)				
Yes	4003 (40.42)	3604 (41.80)	399 (31.17)	<0.001
No	5900 (59.58)	5019 (58.20)	881 (68.83)	
Alcohol drinker (%)				
Yes	7162 (72.32)	6347 (73.61)	815 (63.67)	<0.001
No	2741 (27.68)	2276 (26.39)	465 (36.33)	
Hypertension (%)				
Yes	3605 (36.40)	2718 (31.52)	887 (69.30)	<0.001
No	6298 (63.60)	5905 (68.48)	393 (30.70)	
PIR	2.10 (1.09-4.04)	2.14 (1.09-4.12)	1.82 (1.02-3.32)	<0.001
TCHO (mg/dL)	189 (163-216)	191 (166-218)	173 (148-204)	<0.001
TG (mg/dL)	102 (71-148)	99 (69-145)	122 (87-173)	<0.001
LDL-C (mg/dL)	111 (89-136)	113 (91-137)	97 (74-123)	<0.001
HDL-C(mg/dl)	52 (43-63)	52 (43-64)	47 (40-57)	<0.001
HbA1c (%)	5.50 (5.20-5.90)	5.40 (5.20-5.70)	6.90 (6.20-8.20)	<0.001
NLR	1.91(1.43-2.56)	1.88 (1.41-2.50)	2.12 (1.57-2.87)	<0.001

*Notes* Median (interquartile range) for continuous variables and % for categorical variables. BMI, body mass index; PIR, poverty income ratio; TCHO, Total cholesterol; TG, triglyceride; LDL-C, Low-density lipoprotein cholesterol; HDL-C, High-density lipoprotein cholesterol; HbA1c, glycated hemoglobin A1c; NLR, neutrophil-to-lymphocyte ratio

### The relationship between NLR and T2DM

As shown in Table 2, a significant correlation was identified between NLR and T2DM. Covariates were not adjusted for in Model 1, while Model 2 was adjusted for age, sex, and race; Model 3 included adjustments for all covariates. In conclusion, analyses revealed that in Model 3, NLR remained positively associated with T2DM (OR:1.14,95%CI:1.05–1.24, *P* = 0.003). Subsequently, quartile analysis of NLR was conducted, using Q1 as a reference, and the OR for Q4 was significantly higher than that for Q1 (OR: 1.86, 95% CI: 1.58–2.21, *P* < 0.001). Following complete adjustment for all covariates, Patients in the highest quartile of NLR have a risk of developing the disease that is more than one time higher than those in the lowest quartile (OR: 1.56, 95% CI: 1.19–2.06, *P* = 0.002).

**Table 2:** The association between NLR levels and prevalence of T2DM by logistic regression analyses

		Model 1		Model 2		Model 3	
		OR (95% CI)	*P* - value	OR (95% CI)	*P*- value	OR (95% CI)	*P* - value
NLR		1.27(1.20,1.35)	<0.001	1.20(1.13,1.27)	<0.001	1.14(1.05,1.24)	0.003
NLR (quartile)							
Q1	1.11±0.24	reference		reference		reference	
Q2	1.67±0.14	1.13(0.94,1.36)	0.180	1.18(0.98,1.43)	0.086	1.08(0.83,1.40)	0.566
Q3	2.20±0.19	1.45(1.22,1.73)	<0.001	1.52(1.26,1.83)	<0.001	1.37(1.06,1.77)	0.015
Q4	3.66±1.58	1.86(1.58,2.21)	<0.001	1.76(1.47,2.12)	<0.001	1.56(1.19,2.06)	0.002
*P* for trend		<0.001		<0.001		<0.001	

95% CI: 95% confidence interval

Model 1: no covariates were adjusted

Model 2: adjusted for age, sex, race

Model 3: adjusted for age, sex, race, education level, BMI, smoking status, recreational activities, Alcohol drinker, hypertension, PIR, TCHO, TG, LDL-C, HDL-C, HbA1c

### Subgroup analyses

To ascertain the robustness of the association between NLR and T2DM across various population subgroups, subgroup analyses were conducted following Model 3. Table 3 demonstrates that the interaction effect between NLR and T2DM was statistically significant concerning race and hypertension (*P* < 0.05); in contrast, no significant interactions were observed for age, sex, education level, BMI, smoking status, recreational activities, and alcohol drinker (*P* > 0.05).

**Table 3:** The results of subgroup analyses

Character	OR (95%CI)	*P* value	*P* for interaction
Age			0.057
20-39	1.29(1.02,1.65)	0.037	
40-59	1.12(0.99,1.27)	0.081	
≥ 60	1.29(1.02,1.67)	0.010	
Sex			0.554
Male	1.08(1.01,1.23)	0.030	
Female	1.13(1.03,1.23)	0.008	
Race			0.018
Mexican American	0.97(0.87,1.09)	0.644	
Other Hispanic	1.19(0.95,1.51)	0.135	
Non-Hispanic White	1.13(1.04,1.22)	0.004	
Non-Hispanic Black	1.25(1.07,1.47)	0.005	
Other Race	1.51(1.10,2.08)	0.012	
Education level			0.247
< High school	1.15(1.02,1.30)	0.026	
≥High school	1.09(1.02,1.16)	0.007	
BMI (kg/m^2^)			0.282
<25	1.12(0.98,1.28)	0.095	
25-29.9	1.04(0.96,1.13)	0.301	
≥30	1.16(1.06,1.27)	0.002	
Smoking status			0.939
Never	1.09(1.00,1.19)	0.049	
Former	1.12(1.02,1.22)	0.017	
Current	1.13(0.97,1.30)	0.112	
Recreational activities			0.894
Yes	1.12(0.99,1.26)	0.081	
No	1.10(1.03,1.17)	0.003	
Alcohol drinker			0.373
Yes	1.15(1.06,1.24)	<0.001	
No	1.06(0.98,1.15)	0.171	
Hypertension			0.008
Yes	1.08(1.01,1.15)	0.028	
No	1.20(1.07,1.34)	0.001	

*Notes* Subgroup analysis of the association between NLR and T2DM; BMI = body mass index

### Non-linear association between NLR and T2DM

**Fig. 2:**
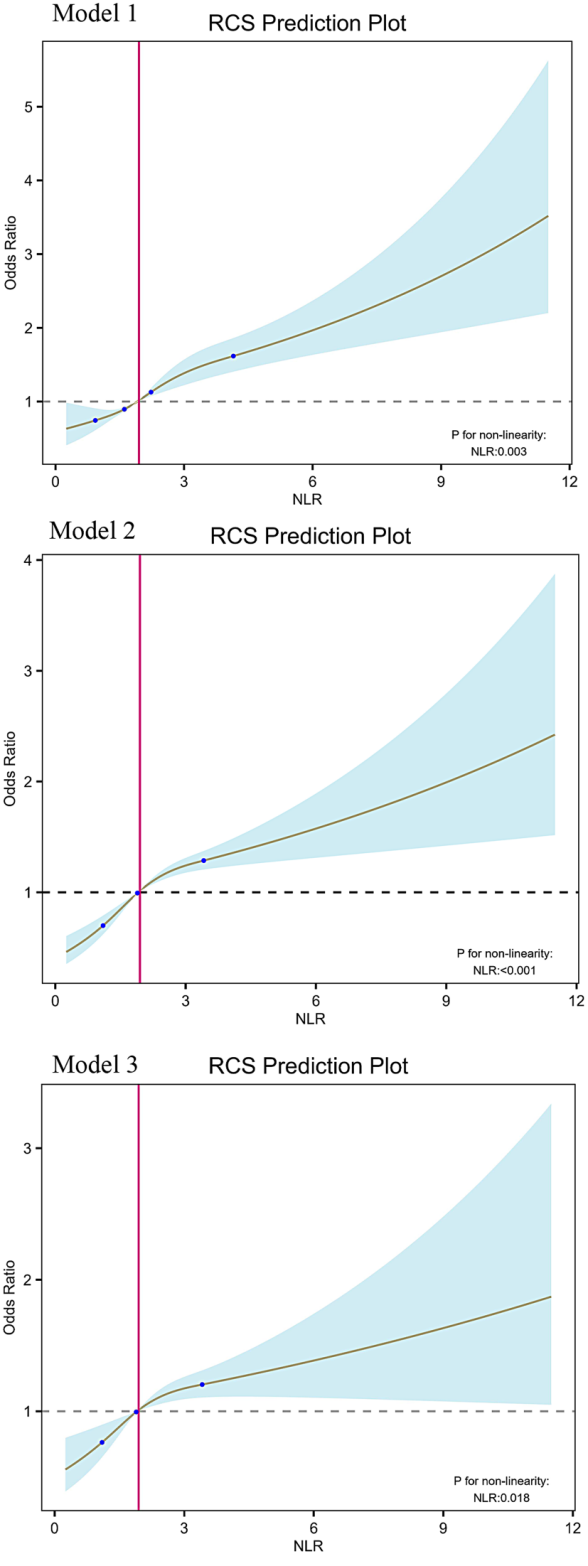
The association between NLR and T2DM. RCS shows a non-linear relationship between NLR and T2DM. The fitted regression line is a solid black line; the black dashed line indicates the position where the OR is equal to 1; the shaded area indicates the 95% CI; NLR, neutrophil-to-lymphocyte ratio

RCS was employed to demonstrate better the relationship between NLR and T2DM (Fig. 2); a strong non-linear correlation was observed between NLR and T2DM,

We conducted a threshold effect analysis and found an inflection point. The inflection points of models 1, 2, and 3 were generally consistent. After adjusting covariates according to Model 3, the inflection point was 2.27. Observations indicate that when NLR is below the inflection point, the risk of T2DM is lower, when NLR exceeds the inflection point, the risk increases rapidly.

## Discussion

In this cross-sectional study, we utilized the NHANES database to analyze relevant data from adult participants in the United States. We explored the relationship between NLR and the risk of T2DM, and we concluded: NLR levels in T2DM patients were significantly higher than those in non-T2DM patients. There was a significant positive correlation between NLR and the risk of T2DM, and this relationship persisted even after adjusting for multiple confounding factors. RCS analysis showed a significant nonlinear relationship between NLR and T2DM, with an inflection point at 2.27. The subgroup analyses revealed a significant interaction effect between NLR and T2DM concerning race and hypertension (*P* for interaction < 0.05). In contrast, no significant interactions were found for age, sex, education level, BMI, smoking status, recreational activities, and alcohol drinker (*P* for interaction > 0.05).

T2DM represents a prevalent endocrine system disorder characterized by multiple metabolic disturbances that induce a state of chronic hyperglycemia [11, 24]. Evidence suggests that low-grade inflammation plays a crucial role in the pathogenesis of T2DM [26], and HbA1c, a commonly used laboratory marker for diagnosing T2DM, reflects the average blood glucose levels of the human body over three months, facilitating the monitoring of these levels [27]. However, HbA1c does not assess changes in the body’s inflammatory state. The NLR can effectively recognize such changes [28]. In clinical practice, blood counts can be easily tested, offering rapid and cost-effective results. NLR, as a ratio of neutrophils to lymphocytes, provides greater accuracy than a single measurement and effectively reflects the systemic inflammatory response [29]. Neutrophils and T-lymphocytes are pivotal in the development and progression of diabetes. It has been reported that hyperglycemia affects the number and function of circulating neutrophils. However, neutrophils in T1DM and T2DM patients exhibit different characteristics, with increased neutrophil counts observed only in T2DM patients [30]. It has been demonstrated that in patients with T2DM, the expression of activation markers on neutrophil membranes differs from that in healthy controls. This is evidenced by a decreased expression of the adhesion molecule LFA-3, increased levels of activation markers such as CD11B and CD66B, and increased adhesion of neutrophils to endothelial cells, leading to systemic inflammation and endothelial damage [31–33].In preclinical models, research has demonstrated that neutrophils can induce the release of IL-1β and neutrophil elastase (NE) through the NF-κB pathway, disrupting insulin signaling and degrading insulin receptor substrate-1 (IRS-1), respectively, thereby contributing to IR [34, 35]. Th17 represents a subset of CD4 + T cells characterized by the secretion of pro-inflammatory factors, including IL-17. Studies have indicated that IL-17 can stimulate the production of TNF-α and is implicated in the development of IR [36]. Regulatory T (Treg) cells constitute a minor subset of T lymphocytes known for their pivotal role in suppressing inflammatory responses [37]. During the progression of diabetes, Treg cells can inhibit Th1 and Th17 cell responses through the regulation of the microenvironment and alteration of surface receptor expression, consequently ameliorating IR [38]. Nonetheless, diabetic patients exhibit a significant reduction in the number of Treg cells [39].

Recently, the NLR has emerged as a novel indicator of inflammation across a spectrum of diseases, with several reports demonstrating its elevation in conditions such as prostate cancer [40], COVID-19 infection [41], and chronic obstructive pulmonary disease [42]. Furthermore, some scholars have extensively studied the application value of NLR in diabetes and its complications. Mohammed et al. conducted a case-control study with 160 T2DM patients and 132 non-T2DM patients to assess the potential role of NLR in predicting T2DM progression and treatment. The results indicated a significant difference in NLR values between T2DM and non-T2DM patients (4.189 ± 4.154 vs. 4.095 ± 8.851, *P* = 0.009). NLR exhibits high sensitivity and specificity, which assists in the management of T2DM and the prediction of long-term complications [43]. Hussain et al. evaluated the relationship between NLR and different levels of glycemic control in T2DM patients, randomly dividing 330 T2DM patients into three groups based on glycemic control. The results showed that higher NLR levels were related to elevated HbA1c and poor glycemic control in T2DM patients. Additionally, NLR can serve as an indicator for monitoring glycemic control in diabetes [44]. Adane et al. explored the relationship between NLR and glycemic control in T2DM patients. Thirteen studies were included, and the results showed a correlation between high NLR values and increased HbA1c in T2DM patients. Hence, NLR should be regarded as a marker of glycemic control in T2DM patients [45]. NLR not only predicts T2DM progression and monitors glycemic control but also plays an important role in identifying diabetic complications. Liu et al. demonstrated that T2DM patients with elevated NLR levels are more likely to develop diabetic peripheral neuropathy. NLR can help doctors understand the progression of diabetic peripheral neuropathy [46]. Li et al. suggested that NLR might be an effective potential inflammatory marker for identifying the risk of diabetic kidney disease in T2DM patients in the United States. T2DM patients with elevated NLR levels should have their potential risk to kidney function closely monitored [47]. Studies have also found that NLR is highly valuable in diagnosing diabetic retinopathy [48]. The above reports indicate that NLR, as an inflammatory marker, has positive potential applications in predicting and managing diabetes and its complications. Our research also confirmed a significant positive correlation between NLR levels and T2DM risk, further demonstrating that NLR could be an effective indicator for predicting early T2DM risk in clinical settings.

Our subgroup analyses revealed that the prevalence of T2DM was higher in women than in men for each unit increase in NLR, and the prevalence of T2DM was higher in the obese population compared to the normal-weight and overweight populations. This sex disparity could be attributed to physiological and psychosocial factors, with women exhibiting greater susceptibility to T2DM than men, particularly in relation to psychosocial stress [49]. Additionally, sex differences have been linked to variations in fat distribution within adipose tissue among both women and men [50]. Obesity represents a pivotal risk factor for the onset of T2DM [51], with the intricate mechanisms bridging the two conditions remaining complex and ambiguous. Numerous studies have demonstrated that adipose tissue releases surplus circulating fatty acids, glycerol, hormones, and pro-inflammatory cytokines, potentially interfering with cellular insulin signaling and exacerbating IR [52]. Additionally, we found that the association between NLR and T2DM remained unaffected by age, sex, education level, BMI, smoking status, recreational activities, and alcohol drinker, indicating that NLR may serve as a reliable predictor of T2DM risk across diverse populations. Nonetheless, the association between NLR and T2DM may vary by race, particularly among Non-Hispanic White, Non-Hispanic Black, and Other Race groups, possibly attributed to differences in body mass across racial demographics. In a cross-sectional study, it was observed that 263 Non-Hispanic Black and Non-Hispanic White adults with lung cancer were included, during which the level of inflammation and prevalence was evaluated using NLR as a biomarker. The results indicated that the level of inflammation and prevalence appeared to be lower in Non-Hispanic Black compared to Non-Hispanic White individuals [53]. This suggests that racial differences may influence NLR levels in inflammatory diseases. Moreover, we employed weighted logistic regression to investigate the relationship between NLR and T2DM, discovering a positive correlation between NLR and T2DM in the general population (OR: 1.27, 95% CI: 1.20–1.35, *P* < 0.001). In Model 3, after adjusting for all covariates, we found that NLR remained significantly associated with T2DM risk (OR: 1.14, 95% CI: 1.05–1.24, *P* = 0.003). This model suggests that for every 1 unit increase in NLR, the risk of developing T2DM increases by 14%. Based on the RCS, we observed that as NLR levels increase, the prevalence of T2DM also rises. When NLR exceeds 2.27, the risk of T2DM increases significantly. The aforementioned research results indicate that NLR can assist doctors in effectively identifying the risk of T2DM, contributing to its prevention and management.

The strength of our study lies in the utilization of a substantial sample size sourced from the NHANES database, and the statistical findings are compelling. Additionally, the NLR serves as a readily accessible laboratory indicator, aiding clinicians in identifying patients at high risk for T2DM. Nonetheless, limitations exist in this current cross-sectional study, including the reliance on participants’ self-reported diagnoses rather than diagnoses confirmed by medical professionals, potentially introducing bias into the results. Furthermore, despite controlling for multiple confounders, the interference of unknown confounders remains a possibility. Lastly, the causal relationship between the NLR and T2DM cannot be established in this study, necessitating further research.

## Conclusion

Our findings indicate a significant positive correlation between neutrophil-to-lymphocyte ratio levels and the risk of T2DM. However, current results cannot determine a causal relationship between the two, further prospective studies are needed to confirm their relationship.

### Electronic supplementary material

Below is the link to the electronic supplementary material.

Supplementary Material 1

## Data Availability

The NHANES holds the data for all analyses conducted throughout the study period and is available upon request. [https://www.cdc.gov/nchs/nhanes/index.htm].

## References

[1] Heald AH, Stedman M, Davies M, Livingston M, Alshames R, Lunt M, Rayman G, Gadsby R (2020). Estimating life years lost to diabetes: outcomes from analysis of National Diabetes Audit and Office of National Statistics data. Cardiovasc Endocrinol Metab.

[2] Harris-Hayes M, Schootman M, Schootman JC, Hastings MK (2020). The role of physical therapists in fighting the type 2 diabetes epidemic. J Orthop Sports Phys Ther.

[3] Cole JB, Florez JC (2020). Genetics of diabetes mellitus and diabetes complications. Nat Rev Nephrol.

[4] Sun H, Saeedi P, Karuranga S, Pinkepank M, Ogurtsova K, Duncan BB, Stein C, Basit A, Chan JCN, Mbanya JC (2022). IDF Diabetes Atlas: Global, regional and country-level diabetes prevalence estimates for 2021 and projections for 2045. Diabetes Res Clin Pract.

[5] The L (2023). Diabetes: a defining disease of the 21st century. Lancet.

[6] Sharma U, Chakraborty M, Chutia D, Bhuyan NR (2022). Cellular and molecular mechanisms, genetic predisposition and treatment of diabetes-induced cardiomyopathy. Curr Res Pharmacol Drug Discov.

[7] Arroyo MN, Green JA, Cnop M, Igoillo-Esteve M. tRNA Biology in the pathogenesis of diabetes: role of genetic and environmental factors. Int J Mol Sci 2021, 22(2). 10.3390/ijms22020496 PMC7825315 33419045

[8] DeFronzo RA, Ferrannini E, Groop L, Henry RR, Herman WH, Holst JJ, Hu FB, Kahn CR, Raz I, Shulman GI (2015). Type 2 diabetes mellitus. Nat Rev Dis Primers.

[9] Mahmoud M, Abdel-Rasheed M (2023). Influence of type 2 diabetes and obesity on adipose mesenchymal stem/stromal cell immunoregulation. Cell Tissue Res.

[10] Galicia-Garcia U, Benito-Vicente A, Jebari S, Larrea-Sebal A, Siddiqi H, Uribe KB, Ostolaza H, Martin C. Pathophysiology of type 2 diabetes Mellitus. Int J Mol Sci 2020, 21(17). 10.3390/ijms21176275 PMC7503727 32872570

[11] Tsalamandris S, Antonopoulos AS, Oikonomou E, Papamikroulis GA, Vogiatzi G, Papaioannou S, Deftereos S, Tousoulis D (2019). The role of inflammation in diabetes: current concepts and future perspectives. Eur Cardiol.

[12] Aktas G. Association between the Prognostic Nutritional Index and Chronic Microvascular complications in patients with type 2 diabetes Mellitus. J Clin Med 2023, 12(18). 10.3390/jcm12185952 PMC10531521 37762893

[13] Misirlioglu NF, Uzun N, Ozen GD, Calik M, Altinbilek E, Sutasir N, Baykara Sayili S, Uzun H. The relationship between Neutrophil-Lymphocyte ratios with Nutritional Status, Risk of Nutritional Indices, Prognostic Nutritional indices and Morbidity in patients with ischemic stroke. Nutrients 2024, 16(8). 10.3390/nu16081225 PMC11054104 38674915

[14] Shiny A, Bibin YS, Shanthirani CS, Regin BS, Anjana RM, Balasubramanyam M, Jebarani S, Mohan V (2014). Association of neutrophil-lymphocyte ratio with glucose intolerance: an indicator of systemic inflammation in patients with type 2 diabetes. Diabetes Technol Ther.

[15] Chen Y, Liu J, Li Y, Cong C, Hu Y, Zhang X, Han Q (2023). The Independent Value of Neutrophil to lymphocyte ratio in Gouty Arthritis: a narrative review. J Inflamm Res.

[16] Basaran E, Aktas G (2024). The relationship of vitamin D levels with hemogram indices and metabolic parameters in patients with type 2 diabetes mellitus. AIMS Med Sci.

[17] Buse Balci S, Aktas G (2022). A Comprehensive Review of the role of Hemogram Derived inflammatory markers in gastrointestinal conditions. Iran J Colorectal Res.

[18] Sahin S, Sarikaya S, Alcelik A, Erdem A, Tasliyurt T, Akyol L, Altunkas F, Aktas G, Karaman K (2013). Neutrophil to lymphocyte ratio is a useful predictor of atrial fibrillation in patients with diabetes mellitus. Acta Med Mediterranea.

[19] Aktas G, Sit M, Dikbas O, Erkol H, Altinordu R, Erkus E, Savli H (2017). Elevated neutrophil-to-lymphocyte ratio in the diagnosis of Hashimoto’s thyroiditis. Rev Assoc Med Bras (1992).

[20] Afsin H, Aktas G. Platelet to lymphocyte and neutrophil to lymphocyte ratios are useful in differentiation of thyroid conditions with normal and increased uptake. Ethiop J HEALTH Dev 2021, 35(3).

[21] Aktas G, Basaran E, Taslamacıoğlu Duman T, Atak B, Kurtkulagi O, Bilgin S, Demirkol M, Kosekli M (2020). Irritable bowel syndrome is associated with novel inflammatory markers derived from hemogram parameters. Family Med Prim Care Rev.

[22] Aktas G. Hematological predictors of novel coronavirus infection. Rev Assoc Med Bras (1992). 2021;67Suppl 1(Suppl 1):1–2. 10.1590/1806-9282.67.Suppl1.20200678 34259763

[23] Hong J, Lian N, Li M (2023). Association between the neutrophil-to-lymphocyte ratio and psoriasis: a cross-sectional study of the National Health and Nutrition Examination Survey 2011–2014. BMJ Open.

[24] Wu K, Chen L, Kong Y, Zhuo JF, Sun Q, Chang J (2024). The association between serum copper concentration and prevalence of diabetes among US adults with hypertension (NHANES 2011–2016). J Cell Mol Med.

[25] DecisionLinnc Core Team. (2023). DecisionLinnc. 1.0. https://www.statsape.com/.

[26] Munoz Garcia A, Juksar J, Groen N, Zaldumbide A, de Koning E, Carlotti F (2024). Single-cell transcriptomics reveals a role for pancreatic duct cells as potential mediators of inflammation in diabetes mellitus. Front Immunol.

[27] American Diabetes Association Professional Practice C: 2 (2024). Diagnosis and classification of diabetes: standards of Care in Diabetes-2024. Diabetes Care.

[28] Sefil F, Ulutas KT, Dokuyucu R, Sumbul AT, Yengil E, Yagiz AE, Yula E, Ustun I, Gokce C (2014). Investigation of neutrophil lymphocyte ratio and blood glucose regulation in patients with type 2 diabetes mellitus. J Int Med Res.

[29] Kissling M, Fritschi N, Baumann P, Buettcher M, Bonhoeffer J, Naranbhai V, Ritz N (2023). ProPaed, team cs: Monocyte, lymphocyte and Neutrophil Ratios - Easy-to-use biomarkers for the diagnosis of Pediatric Tuberculosis. Pediatr Infect Dis J.

[30] Giovenzana A, Carnovale D, Phillips B, Petrelli A, Giannoukakis N (2022). Neutrophils and their role in the aetiopathogenesis of type 1 and type 2 diabetes. Diabetes Metab Res Rev.

[31] Huang J, Xiao Y, Zheng P, Zhou W, Wang Y, Huang G, Xu A, Zhou Z (2019). Distinct neutrophil counts and functions in newly diagnosed type 1 diabetes, latent autoimmune diabetes in adults, and type 2 diabetes. Diabetes Metab Res Rev.

[32] Pezhman L, Tahrani A, Chimen M (2021). Dysregulation of leukocyte trafficking in type 2 diabetes: mechanisms and potential therapeutic avenues. Front Cell Dev Biol.

[33] Munteanu C, Rotariu M, Turnea MA, Anghelescu A, Albadi I, Dogaru G, Silisteanu SC, Ionescu EV, Firan FC, Ionescu AM (2022). Topical reappraisal of molecular pharmacological approaches to endothelial dysfunction in diabetes Mellitus Angiopathy. Curr Issues Mol Biol.

[34] Watanabe Y, Nagai Y, Honda H, Okamoto N, Yanagibashi T, Ogasawara M, Yamamoto S, Imamura R, Takasaki I, Hara H (2019). Bidirectional crosstalk between neutrophils and adipocytes promotes adipose tissue inflammation. FASEB J.

[35] Ushakumari CJ, Zhou QL, Wang YH, Na S, Rigor MC, Zhou CY, Kroll MK, Lin BD, Jiang ZY. Neutrophil Elastase Increases Vascular Permeability and Leukocyte Transmigration in Cultured Endothelial Cells and Obese Mice. *Cells* 2022, 11(15). 10.3390/cells11152288 PMC9332277 35892585

[36] Xia C, Rao X, Zhong J (2017). Role of T lymphocytes in type 2 diabetes and diabetes-Associated inflammation. J Diabetes Res.

[37] Goldmann O, Nwofor OV, Chen Q, Medina E (2024). Mechanisms underlying immunosuppression by regulatory cells. Front Immunol.

[38] Zhou T, Hu Z, Yang S, Sun L, Yu Z, Wang G. Role of Adaptive and Innate Immunity in Type 2 Diabetes Mellitus. *J Diabetes Res* 2018, 2018:7457269. 10.1155/2018/7457269 PMC6250017 30533447

[39] Yu W, Li C, Zhang D, Li Z, Xia P, Liu X, Cai X, Yang P, Ling J, Zhang J et al. Advances in T Cells Based on Inflammation in Metabolic Diseases. *Cells* 2022, 11(22). 10.3390/cells11223554 PMC9688178 36428983

[40] Salciccia S, Frisenda M, Bevilacqua G, Viscuso P, Casale P, De Berardinis E, Di Pierro GB, Cattarino S, Giorgino G, Rosati D (2024). Prognostic role of platelet-to-lymphocyte ratio and neutrophil-to-lymphocyte ratio in patients with non-metastatic and metastatic prostate cancer: a meta-analysis and systematic review. Asian J Urol.

[41] Sayed AA. Assessing the diagnostic values of the neutrophil-to-lymphocyte ratio (NLR) and systematic Immunoinflammatory Index (SII) as biomarkers in Predicting COVID-19 severity: a Multicentre comparative study. Med (Kaunas) 2024, 60(4). 10.3390/medicina60040602 PMC11052014 38674248

[42] Cai C, Zeng W, Wang H, Ren S (2024). Neutrophil-to-lymphocyte ratio (NLR), platelet-to-lymphocyte ratio (PLR) and monocyte-to-lymphocyte ratio (MLR) as biomarkers in diagnosis evaluation of Acute Exacerbation of Chronic Obstructive Pulmonary Disease: a retrospective, observational study. Int J Chron Obstruct Pulmon Dis.

[43] Mohammed AM, Khaleel M, Jalily RMP, Dhanekula QA, Dinesh Eshwar K (2024). Neutrophil-to-lymphocyte ratio as a potential biomarker to managing type 2 diabetes Mellitus and Predicting Disease Progression. Cureus.

[44] Hussain M, Babar MZM, Akhtar L, Hussain MS (2017). Neutrophil lymphocyte ratio (NLR): a well assessment tool of glycemic control in type 2 diabetic patients. Pak J Med Sci.

[45] Adane T, Melku M, Worku YB, Fasil A, Aynalem M, Kelem A, Getawa S. The Association between Neutrophil-to-Lymphocyte Ratio and Glycemic Control in Type 2 Diabetes Mellitus: A Systematic Review and Meta-Analysis. *J Diabetes Res* 2023, 2023:3117396. 10.1155/2023/3117396 PMC10257553 37305430

[46] Liu S, Zheng H, Zhu X, Mao F, Zhang S, Shi H, Li Y, Lu B (2017). Neutrophil-to-lymphocyte ratio is associated with diabetic peripheral neuropathy in type 2 diabetes patients. Diabetes Res Clin Pract.

[47] Li X, Wang L, Liu M, Zhou H, Xu H (2023). Association between neutrophil-to-lymphocyte ratio and diabetic kidney disease in type 2 diabetes mellitus patients: a cross-sectional study. Front Endocrinol (Lausanne).

[48] Li J, Wang X, Jia W, Wang K, Wang W, Diao W, Ou F, Ma J, Yang Y (2024). Association of the systemic immuno-inflammation index, neutrophil-to-lymphocyte ratio, and platelet-to-lymphocyte ratio with diabetic microvascular complications. Front Endocrinol (Lausanne).

[49] Kautzky-Willer A, Harreiter J, Pacini G (2016). Sex and gender differences in risk, pathophysiology and complications of type 2 diabetes Mellitus. Endocr Rev.

[50] de Ritter R, de Jong M, Vos RC, van der Kallen CJH, Sep SJS, Woodward M, Stehouwer CDA, Bots ML, Peters SAE (2020). Sex differences in the risk of vascular disease associated with diabetes. Biol Sex Differ.

[51] Grant B, Sandelson M, Agyemang-Prempeh B, Zalin A (2021). Managing obesity in people with type 2 diabetes. Clin Med (Lond).

[52] Al-Goblan AS, Al-Alfi MA, Khan MZ (2014). Mechanism linking diabetes mellitus and obesity. Diabetes Metab Syndr Obes.

[53] Byrne CA, Gomez SL, Kim S, Oddo VM, Koh TJ, Fantuzzi G (2022). Disparities in inflammation between non-hispanic black and white individuals with lung cancer in the Greater Chicago Metropolitan area. Front Immunol.

